# Impact of a peer-review network on the quality of inpatient low secure mental health services: cluster randomised control trial

**DOI:** 10.1186/s12913-018-3797-z

**Published:** 2018-12-22

**Authors:** Lina Aimola, Sarah Jasim, Neeraj Tripathi, Paul Bassett, Alan Quirk, Adrian Worrall, Sarah Tucker, Samantha Holder, Mike J. Crawford

**Affiliations:** 1 The Centre for Psychiatry, Imperial College London, London, W12 0NN 759 Q3Q4 UK; 2 The Royal College of Psychiatrists’ College Centre for Quality Improvement, 21 Prescot Street, London, E1 8BB UK; 3 Cygnet Hospital Stevenage, Graveley Road, Stevenage, SG1 4YS UK; 4 Statsconsultancy Ltd, Amersham, HP7 9EN UK; 5 Institute of Group Analysis, 1 Daleham Gardens, London, NW3 5BY UK

**Keywords:** Peer-review networks, Quality improvement, Randomised trial, Low secure services, Forensic mental health

## Abstract

**Background:**

Peer-review networks aim to help services to improve the quality of care they provide, however, there is very little evidence about their impact. We conducted a cluster randomized controlled trial of a peer-review quality network for low-secure mental health services to examine the impact of network membership on the process and outcomes of care over a 12 month period.

**Methods:**

Thirty-eight low secure units were randomly allocated to either the active intervention (participation in the network *n* = 18) or the control arm (delayed participation in the network *n* = 20). A total of 75 wards were assessed at baseline and 8 wards dropped out the study before the data collection at 12 month follow up. The primary outcome measure was the quality of the physical environment and facilities of the services. The secondary outcomes included: safety of the ward, patient mental wellbeing and satisfaction with care, staff burnout, training and supervision. We hypothesised that, relative to control wards, the quality of the physical environment and facilities would be higher on wards in the active arm of the trial 12 months after randomization.

**Results:**

The difference in the primary outcome between the groups was not statistically significant (4.1; 95% CI [− 0.2, 8.3] *p* = 0.06). The median number of untoward incidents rose in control services and remained the same at the member of the network (Difference between members and non-members = 0.55; 95% IC [0.29, 1.07] *p* = 0.08). At follow up, a higher proportion of staff in the active arm of the trial indicated that they felt safe on the ward relative to those in the control services (*p* = 0.04), despite reporting more physical assaults (*p* = 0.04). Staff working in services in the active arm of the trial reported higher levels of burnout relative to those in the control group. No difference was seen in patient outcomes.

**Conclusions:**

We did not find evidence that participation in a peer-review network led to marked changes in the quality of the physical environment of low secure mental health services at 12 months. Future research should explore the impact of accreditation schemes and examine longer term outcomes of participation in such networks.

**Trial registration:**

ISRCTN79614916. Retrospectively registered 28 March 2014.

## Background

A number of different programmes and initiatives have been developed to help clinical teams assess and improve the quality of care they provide. However, evidence for their effectiveness is generally weak and most are promoted on the basis of very limited information about their impact [1].

Many programmes involve peer-review networks in which each service member of the network hosts a multidisciplinary team of peers coming from the other service members to collect evidence about whether care is being delivered according to agreed standards. The multidisciplinary team will then provide feedback on areas of challenge and suggestions for service improvement to the host team [2, 3]. A peer is someone usually in the same branch of health care provision, with comparable experience or training to the staff members working in the host service part of the same network [4].

The setting up of a new peer-review network starts with the development of national standards of care that are based on recommendations made by professional bodies, organisations such as the National Institute for Health and Clinical Excellence, front-line clinicians, service users and carers [5, 6]. Services that choose to participate and become members of the network are required to engage actively in a peer-review cycle consisting of a self-review where they assess themselves against the national standards, a peer-review visit to receive the external feedback on the quality of care they provide and attendance of annual forum to share good practice with all the service members of the network. These activities encourage services to find solutions to the challenges they all share, spread good practice and promote change to improve the quality of care they offer to patients [7].

One of the conceptual models in the literature that links network’s membership and services’ quality of care suggests that through a process of self-review against consensus standards, independent assessment and feedback on performance and sharing examples of good practice, services can increase the quality of care they provide with the expectation that it will lead to the delivery of services that are safe and clinically effective [8, 9].

Peer-review networks have been developed across many areas of healthcare from cancer, chronic obstructive pulmonary disease, coronary heart disease to mental health with the aim to improve service members’ quality of care [10–15]. Longitudinal data collected from services that take part in these networks show that their performance against standards of care generally improves over time [16–20]. However, such studies do not provide a basis for determining the extent to which participation in networks is responsible for these changes and experimental studies have rarely been conducted. Evidence available on the impact of peer-review networks is inconclusive [19, 21]. Moreover, the impact of these programmes on patient outcomes after they are established, is largely unexplored and evidence about whether they have an impact on patient outcomes is needed [22].

The Royal College of Psychiatrists’ College Centre for Quality Improvement (CCQI) is one of the largest providers of external peer-review programmes in the UK. The peer-review networks that the CCQI hosts aim to improve the quality of care that people with poor mental health receive across a wide range of psychiatric settings [23]. In 2006 the College set up a peer-review network for inpatient forensic Medium Secure Mental health services (MSU) which provide inpatient treatment for adults detained under the Mental Health Act (1983) with severe mental health disorders who pose a significant risk of harming themselves or others. Following a thorough risk assessment, forensic patients can be admitted to High, Medium or Low Secure Forensic Mental Health Services. Each level of risk along this spectrum will require a specific range of physical, procedural and relational security measures to provide effective care treatment and assure safety for the patients and others [24]. The development of the MSU network at the CCQI helped service members to identify areas of good practice as well as areas for improvement by promoting a culture of openness and enquiry between peers. During the first wave of peer-review visits it became apparent that there was substantial variation in the quality of the physical environment of the services. The features of the physical environment in forensic services should comply with the primary functions of safety, therapy and security in line with the needs as well as the risk profile of the patients. Examples of these features include a perimeter, airlock, seclusion facilities, de-escalation room, absence of ligature points and a range of occupational facilities to help supporting rehabilitation and sustainable discharge into the community. In subsequent cycles of the MSU, successful efforts were made to provide an environment that was safer and in line with the recommended standards of care [25–27].

When a decision was made to extend this network to low secure mental health services, we took the opportunity to examine the impact of membership of the new network. Low secure services have much in common with medium secure services, however the level of physical security offered is lower and patients are able to access a wider range of services aimed at preparing them for discharge into the community. We set out to examine whether quality of care in services that took part in the peer-review network was higher than in those that did not 1 year it was established. Our primary hypothesis was that, 12 months after joining the peer-review network, member services would have a better quality of physical environment and facilities than services that were randomly allocated to a wait list control. We also examined the impact of network membership on safety on the ward, patient-rated satisfaction with care and mental wellbeing, staff burnout, training and supervision.

## Methods

### Study design and sample

Details of the design and methods of the trial are reported elsewhere [28]. In summary, we conducted a single-blind, parallel-group, cluster-randomised controlled trial, of the impact of membership of a peer-review network on the quality of inpatient low secure mental health services. Staff managing the quality network attempted to contact all providers of low secure inpatients services in England and Wales to enquire whether they wanted to take part in the study. All low secure services were eligible to take part in the trial aside from those that were connected to a medium secure service that was already participating in a peer-review network. We excluded these services as they may already have had some exposure to the intervention being tested. Details of services that agreed to take part in the study were sent to an independent team that randomised them using a web-based randomisation service (http://www.random.org) and a randomisation ratio of 1:1. Randomisation was stratified according to the size of the service (whether they contained up to four, or more than four wards). Services were allocated to either the active arm (participation in the peer-review network plus quality improvement as usual) or the control arm of the trial (quality improvement as usual plus delayed participation in the network). All services in the study continued to undertake local improvement initiatives such as clinical audit, and national initiatives such as inspections from statutory authorities.

Following randomisation, services were notified of their allocation status and arrangements were made for a researcher to collect baseline data. Prior to data collection, staff members at each service were repeatedly reminded that the researcher collecting study data must not be told which arm of the trial they were in. In the event of accidental unmasking of a researcher, all further data were collected by a second researcher who was masked to the allocation status of the unit.

### Interventions

Services allocated to the active arm of the trial all received a welcome pack within 2 weeks of being randomised. The welcome pack included information about the network and a series of checklists that allowed staff working in the service to indicate the extent to which they were meeting recommended standards of care.

Services received their self-review pack 12 weeks before their scheduled peer-review date and were required to submit the completed self-review documents 4 weeks before the review. At the peer-review visits, members of the review team were given an escorted tour of the service to assess the patients’ facilities, they met with current service users to talk to them about the care they received and they reviewed policy documents kept by the service. Members of the review team then conducted interviews with senior managers, and front-line staff working in the service. At the end of the visit, the review team provided feedback to managers and staff.

representatives to highlight discrepancies between the self-assessment and the peer-review data, and summarise the service’s achievements as well as areas that need improvements. After the peer-review stage, each service is provided with a local report that compiles self and peer-review data and shows the extent to which the service meets the standards. When all member services had completed the peer-review phase, an annual national report of the aggregated findings is written to enable benchmarking with other services and reflect on their practice. The final stage of the review cycle involves action planning, an invitation to an annual forum and access to newsletters and an e-mail discussion group. At the annual forum members of the network have an opportunity to hear about challenges faced by similar units and share examples of how different units have tried to meet these challenges.

All services in the control arm of the trial were free to carry out any other quality improvement initiatives as per normal. These may include local reviews, audits and the possibility of inspection by statutory bodies. Twelve months after randomization, all control services were invited to join the quality improvement network.

### Outcomes

The primary outcome was the quality of the physical environment and facilities on the wards which was assessed at ward-level using the Quality of Environment in Low Secure Services checklist (QELS) [29]. The QELS checklist assesses 10 domains of quality of care and generates a total score of zero to 100, with higher scores indicating a higher quality of environment. Previous research has demonstrated that the QELS has good inter-rater reliability and concurrent validity [29]. The checklist was completed at baseline and follow-up by a member of the research team who visited each ward to collect the study data. During these visits the researcher was always masked to allocation status of the unit.

The secondary outcomes were safety on the ward, patient satisfaction with care and mental wellbeing, staff burnout, training and supervision. Safety on the ward was assessed using ward-level data and information from staff and patients about safety. At ward-level safety was measured by recording the number of untoward incidents during the previous 6 months obtained from records kept by the service. An untoward incident was defined as an event or circumstance that could have resulted, or did result, in unnecessary damage, loss or harm such as physical or mental injury to a patient, staff, visitors or members of the public [30]. The type of untoward incidents that we recorded referred to: police involvement, damage to property, physical assault, verbal abuse, self-harm, substance misuse, pharmacy (e.g. dispensing the wrong drug), security, fire, slips and trips. Information on safety was gathered from patients and staff via a staff and patient questionnaire, which included two questions on safety: whether they had been assaulted in the previous 3 months - yes or no -(“physical safety”) and whether they felt safe on the ward, using a four point Likert scale, zero – never, to four – always (“emotional safety”).

Patient satisfaction with care was assessed using a modified version of the Patient Satisfaction Questionnaire (PSQ) [31]. The questionnaire consisted of four questions, one of which was modified following the approval of the authors by changing one item to make it suitable for the inpatient setting. Patient mental wellbeing was assessed with the Short Warwick-Edinburgh Mental Well-being Scale (SWEMWBS) [32, 33]. Higher scores on this questionnaire indicated a higher level of mental wellbeing. The questions on both satisfaction with care and mental wellbeing were included in the patient questionnaire along with the questions on safety outlined above. Staff burnout was measured using the Maslach Burnout Inventory (MBI) which is a validated measure of burnout used extensively in human services, education and business [34, 35]. The MBI was included in the staff survey which, was distributed to staff by the visiting researcher. The staff questionnaire also included questions on whether the training and the clinical supervision they receive was adequate to carry out their job.

### Procedures

Services in the active arm of the trial joined the quality network immediately, whilst those in the control arm joined the network 12 months later. Services in the active arm of the trial completed a self-review, had a visit from a peer-review team and received a local report based on the peer-review’s findings. During the self-review phase, services scored themselves against a range of nationally agreed standards of care developed to help the implementation of the Department of Health’s recommendations on good practice for forensic services [36]. In the peer-review phase, visiting staff members from other low secure services assessed the extent to which the host unit complied with those standards and discussed any discrepancies with the self-assessment. At the end of the peer-review visit, the review team provided initial verbal feedback to the host team about the strengths and weaknesses of the care they provide. After the peer-review visit, the host service received a detailed local report summarising their performance against each standard reviewed. Each service also received a copy of a national report which allowed them to benchmark themselves against other services and reflect on their performance. In addition to this, each service in the active arm of the trial was asked to nominate members of their team to become peer reviewers and were invited to attend an Annual Forum to facilitate the sharing of good practice amongst the service members. Services in the active arm of the trial were also the opportunity to exchange ideas through newsletters, email discussion groups and workshops that are offered to members of the network.

Baseline and follow-up data were gathered by a researcher masked to allocation status via direct observation of each individual ward and its facilities, interviews with the ward manager (to collate routine data on untoward incidents) and the staff and service user survey. During the data collection visits, the researcher engaged with staff and patients by providing further details about the study and sought their informed consent to participate. All service users were asked to take part in the survey, except those where clinical staff judged that it was not appropriate at the time of the visit. Participants were given the option of completing and returning the survey in a sealed envelope on the day of the assessment to the visiting researcher or to post it back to the research team. We did not collect any demographic information on the participating patients and staff and the questionnaire they completed were kept anonymous. This was done to encourage the participants to provide a truthful feedback on the quality of care they received (in the case of the patients) and on their experience of working in the services (in the case of the staff members) and also to increase participation in the study. Generally, the wards of the participating services catered for people with severe mental illness predominately with schizophrenia, non-affective psychosis, personality disorders, learning disabilities and autism spectrum disorder including both men and women from 18 to 65 years old. All data collected at baseline were re-administrated 12 months later at follow-up.

### Data management and analysis

Using a 5% significance level and 90% power, we calculated that a sample of 60 wards was required taking into account a 10% drop out. Assuming no change in the primary outcome in the control arm, the sample size was based on detecting a 10% difference between groups, equivalent to 10 units of the environmental checklist score. Data were analysed using Stata (version 13.1). All primary outcome data and 10% of each secondary outcome measure were double checked against source data for accuracy. The error rate observed for all measures was lower than the 5% set above which all data in the dataset would be double-entered. No double data entry was therefore needed. Descriptive characteristics of the study groups were summarised as either mean (SD), median (IQR), or numbers and proportions as appropriate (Table 1).

**Table 1:** Summary of the results for the ward-, patient- and staff-level outcome measures

Outcome	Baseline		12 month follow-up adj^a^		Adjusted difference at 12 m (95% CI)	*p*-value
	Network	Control	Network	Control		
Ward level outcome						
Quality of Environment in Low secure Services (QELS) – mean (SD)	64.0 (14.6)	65.8 (14.8)	74.0	69.9	4.1 (−0.2, 8.3)^b^	0.06
Untoward incidents - median (IQR)^c^	31 (12, 58)	57 (23, 161)	31 (23, 50)	87 (35, 192)	0.55 (0.29, 1.07)^b^	0.08
Patients outcomes						
Short Warwick-Edinburgh Mental Well-being (SWEMWB) – mean (SD)	24.4 (5.7)	24.5 (5.6)	23.9 (5.6)	23.7 (6.0)	0.4 (−1.1, 2.0)^d^	0.58
Patient Satisfaction Questionnaire (PSQ) – mean (SD)	11.1 (3.8)	11.8 (3.4)	10.9 (3.8)	11.3 (3.6)	0.3 (−0.8, 1.2)^d^	0.60
Physical safety – number reporting an assault (%)	27 (17%)	40 (15%)	37 (18%)	40 (16%)	0.88 (0.40, 1.95)^d, e^	0.42
Emotional Safety (N, N %)^f^	75 (46%)	105 (39%)	95 (47%)	102 (41%)	0.91 (0.54, 1.52)^d, e^	0.71
Staff outcomes						
Maslach - Emotional exhaustion scale	15.1 (11.3)	16.6 (11.7)	17.1 (11.6)	16.5 (11.1)	1.9 (−0.2, 3.9)^d^	0.07
Maslach - Depersonalisation scale	3.1 (3.8)	4.2 (5.1)	4.5 (4.9)	4.2 (4.5)	1.1 (0.3, 2.0)^d^	0.007
Maslach - Personal accomplishment scale	34.5 (8.3)	33.9 (9.3)	35.5 (8.1)	35.3 (8.6)	−0.6 (−2.1, 1.0)^d^	0.49
Physical safety – number reporting an assault (%)	60 (15%)	172 (30%)	81 (18%)	140 (27%)	1.72 (1.04, 2.84)^d, e^	0.04
Emotional Safety (N, N %)^f^	170 (42%)	253 (44%)	208 (47%)	231 (44%)	1.44 (1.02, 2.03)^d, e^	0.04

^a^ Follow-up scores adjusted for baseline. Figures presented for the QELS are marginal means

^b^ Difference adjusted for values at baseline reported as the Network minus the Control group

^c^ Analysis performed with variable on log scale. Difference represents the ratio of outcome in the Network group relative to the Control group

^d^ Difference assessed by time by group interaction. Reported as the Network minus Control group

^e^ Odd ratios

^f^ Summary statistics are the number/percentage of subjects responding either Sometimes or Always

All analyses followed the intention to treat principle. The primary analysis compared mean scores between study groups on the QELS checklist at 12 month follow up. A feature of the data was that some sites had multiple wards included in the study. It was likely that outcomes from wards within the same site would be more similar relative to wards from differing sites. Therefore, to take this into account, the analyses were performed using multilevel methods [37]. The primary outcome measure data were collected at ward-level, therefore, two-level models were used, with wards nested within sites. This outcome was continuous in nature, and found to be normally distributed. As a result, the analysis was performed using multilevel linear regression. The primary analysis was run on the values at follow-up and the baseline scores were used as covariate.

To analyse the survey data on physical and emotional safety, three-level models were used, with service users/staff contained within wards, which were in turn nested within sites. The approach used to analyse these data was to pool the baseline and follow-up data, and to include terms in the analysis for time of measurement (baseline or follow-up) and study group (control or intervention). The effect of the intervention was assessed by examining the size of the time by group interaction. Odds ratios are presented as a measure of the difference in outcome between groups (with corresponding confidence intervals). For the binary outcome (“physical” safety), these reflect the odds of a “yes” response at follow-up in one group relative to the other, after adjusting for baseline differences. For “emotional” safety (which was assessed on a five-point scale), the odds ratios represent the odds of being in the next highest category for one group relative to the other. A significant interaction would imply that group difference varied between time points (i.e. that differences at follow-up were different to those observed at baseline).

We used a three-level model to analyse data from the patient and staff survey with staff/service users contained within wards, which were in turn nested within sites. The approach used to analyse these data was to pool the baseline and follow-up data, and to include terms in the analysis for time of measurement (baseline or follow-up) and study group (control or intervention). The effect of the intervention was assessed by examining the size of the time by group interaction. Finally, further analyses were carried out to compare between the study groups in terms of the patient-level measures relating to satisfaction with care (PSQ) and mental wellbeing (SWEMW) as well as staff-level measures on burnout, training and supervision For these continuous outcomes, the group difference is assessed by the size of the time by group interaction. This is a measure of the mean difference between groups at follow-up, adjusted for baseline differences at follow-up. Scores from the Maslach Burnout Inventory were considered on a continuous scale and separate analysis were carried out for each of the three subscales of the questionnaire: Emotional exhaustion, Depersonalisation and Personal accomplishment.

Ethical approval for the study was obtained from the Royal College of Psychiatrists’ Ethics Committee prior to the start of data collection (Reference number: 2012–3). The study was registered with Current Controlled Trials (ISRCTN79614916).

## Results

**Fig. 1:**
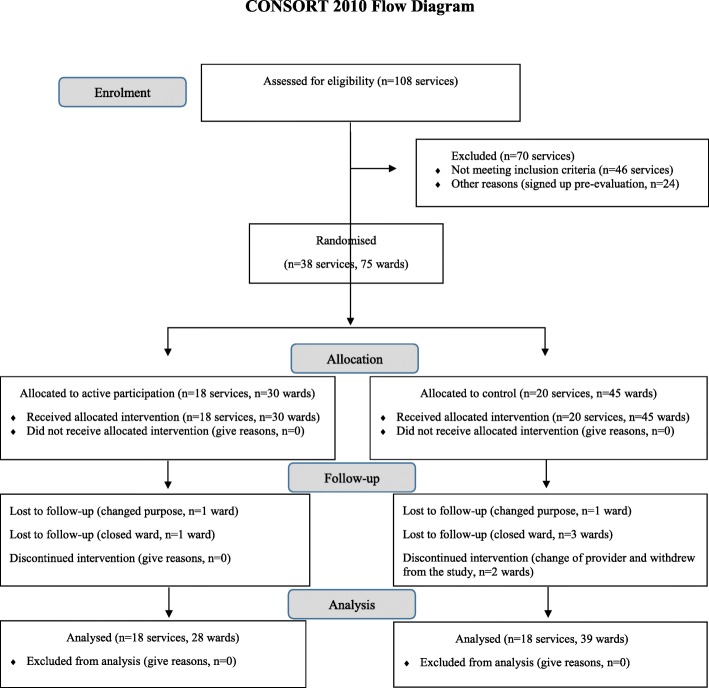
Number of services (and wards within those services) at baseline and follow up

Between June 2012 and June 2014, 38 services (made up of 75 wards) were recruited to take part in the trial. Eighteen (30 wards) were randomly allocated to participation in the network and 20 (45 wards) were allocated to the wait list control (Fig. 1). Eight (11%) wards dropped out the study before the collection of 12-month follow-up data. Two were temporarily closed for refurbishment, two closed down completely, two changed their function and ceased to operate as low secure services and two were taken over by another provider who withdrew them from the study. Thirty-three services had up to 3 wards and the remaining 5 had between 4 and 6 wards. The patients’ mean length of stay in months was 22.10 (SD = 13.03) at baseline and 28.57 (SD = 21.36) at follow-up.

All services that were allocated to the network completed a self-review (within a median of 28 weeks (range 45) following randomisation), and hosted a peer-review visit (within a median of 34 weeks (range 45) following randomisation). Staff at 11 (61%) services undertook training to become a reviewer and 14 (78%) sent at least one staff member to the annual forum. All services received a detailed local report on their strengths and areas for improvement within a median of 6 weeks (range 6) period of completion of the visit. Service members also received a national report to benchmark themselves with other participating services.

The researcher who was due to collect 12-month follow-up data was unmasked on nine occasions (24% of services). For all nine services, a second masked researcher collected all follow-up data.

At baseline a total of 438 (56.3%) patients completed the survey. At follow-up 459 (62%) patients completed the survey. There was no difference in the response rate to survey between study arms (59.9% active arm and 60.6% control arm of the trial).

### Primary and secondary outcomes

The mean total score on the QELS checklist at baseline was 65.2 (SD = 14.5) and the areas in greatest need of improvement were in the provision and quality of seclusion rooms, de-escalation areas and facilities needed to deliver occupational activities.

Baseline and follow-up outcome scores by trial arm are presented in Table 1. There were no differences between the groups at baseline or at follow up across all variables [*p* > .05]. However, at baseline a higher number of staff in the control group indicated that have been physically assaulted within the 3 months prior completing the survey. The mean difference (adjusted for baseline) at follow-up between groups is reported, along with corresponding confidence intervals. The ICC value for the follow-up scores was 0.79 when unadjusted for the baseline value and 0.76 after adjusting for the baseline score suggesting a high degree of clustering by site. Over the 12 month follow-up period there was a mean increase in QELS score of 10.0 in services that took part in the network compared to 4.1 in the control arm. There was a baseline-adjusted mean difference of 4.1 at 12 months, between groups, a difference which almost of statistical significance (*p* = 0.06).

The median number of reported untoward incidents in services in the control arm of the study increased during the follow-up period, but remained the same in services in the network. The number of untoward incidents in the control arm of the trial was almost twice that found in services in the active arm of the trial, but the difference was not statistically significant (*p* = 0.08).

No differences were seen in patient-level outcomes at 12 months.

Regarding outcomes for staff, scores on the Depersonalisation scale of the Maslach Burnout Inventory were higher at follow-up amongst staff of the service members relative to those in the control group (*p* = 0.007). No difference between the groups was observed at follow-up with regard to the Personal Accomplishment or Emotional Exhaustion scale.

Staff working at services allocated to the network reported feeling more safe on the ward compared to those working at services in the control arm of the trial (*p* = 0.04). However, staff in the service members were also more likely to report physical assault episodes in the previous 3 months (*p* = 0.04).

We did not find evidence of differences between the two study groups at follow-up with regard to staff training or the quality of clinical supervision received.

## Discussion

In this cluster randomised controlled trial we investigated whether joining a peer-review network improved the quality of the physical environment, safety, and patient outcomes in low secure services relative to a waitlist control. We also explored whether network membership had any impact on staff burnout, training and supervision. We did not observe any difference between the study groups in relation to the quality of the physical environment or the number of untoward incidents at 12-month follow up. However, it was worth noting that over 12 months, the low secure services that undertook the peer-review process showed a trend towards greater improvement in the quality of the physical environment relative to the non-members. In addition, low secure services in the active arm of the trial were able to contain the number of untoward incidents over the follow-up period while the number of these incidents rose in non-members services during this period. As for patient experience and outcome measures, we did not find any evidence that membership of the network affected patient satisfaction with care or mental wellbeing. In contrast, participating in the peer-review network was associated with differences in staff experience. While staff working in services that joined the network indicated that they felt safe on the ward they also reported higher levels of burnout compared to staff working in services in the control arm of the trial.

This study has several strengths. It is the first randomised evaluation of a peer-led network for mental health services and the first time that any randomised evaluation of a peer-review network has attempted to examine patient reported outcome measures. Study data were collected by independent researchers who were blind to the allocation status of services. Our primary outcome measure provided a reliable and valid measure of a number of key aspects of physical environment and facilities provided by low secure services [29].

There are a number of possible explanations for the broadly negative results we found. Firstly, it is possible that the introduction of this peer network into healthcare system that was already using a range of other methods for assessing and improving service quality, did not add to the overall quality of the service. An alternative explanation is that, we collected follow-up data too soon for the benefits of membership of the network to be seen [12, 13]. At the point which we started the study, there was pressure on all low secure services to join the network and a follow-up period of longer than 1 year was considered unacceptable. However this meant that staff had less than 6 months to make changes to their service between receiving feedback from peer-reviewers and the collection of 12- month follow-up data. This length of time may be insufficient for teams to have implemented recommendations made by peer reviewers. In a study by Roberts and colleagues which examined the impact of participation in a peer-led quality improvement initiative for patients admitted to general hospitals with acute Chronic Obstructive Pulmonary Disease (COPD), the authors found very little difference between the hospitals that undertook the peer-review process and those that did not at 1 year [12]. When outcomes between services were compared 3 years later, the team found evidence of greater improvement in services that received peer feedback [13].

Recent findings from a qualitative study examining the process and outcomes of peer-review networks for inpatient mental health services reported that front line staff do not initially understand or feel engaged with the peer-review process and it may take some time for them to fully use the opportunities for sharing good practice with colleagues from other services [38]. These findings add weight to the possibility that the 12-month follow-up we used in the trial was insufficient to find evidence of service improvement associated with membership of the network.

While we cannot rule out the possibility of some contamination between treatment arms, we believe that the absence of marked improvements in service quality at control sites makes this an unlikely explanation for the negative results of the trial [39]. Although none of the services in the control arm of the trial had a peer-review visit or received a copy of a local or national report, standards for the network could be accessed on the web. Services in the control arm of the trial knew that they would be taking part in the network after 12-month follow data had been collected, and managers at such services may have used the published standards for low secure in preparation for their first self-review.

In this study we focussed on components of care that are more easily quantifiable such as the quality of the physical environment. Feedback collected from those taking part in the network suggested that their involvement helped them develop more effective ways of working as a team and made them more open to feedback from others [38]. Such outcomes may help improve service quality in ways that we did not measure in this trial.

Caution also needs to be used when interpreting data on staff and patient outcomes. This is because there was turnover of both patients and staff between the baseline and follow-up assessments. This meant that we were unable to compare patient mental health and satisfaction and staff burnout at an individual level. We estimate that half the inpatients and a quarter of staff working on the units at the time of the baseline assessment were not at these units when 12-month follow-up data were collected. It is therefore possible that the absence of change in patient outcomes and differences in staff outcomes reflect differences in the makeup of these groups between baseline and follow-up, rather than real changes associated with the active intervention we studied.

Nonetheless, higher levels of depersonalisation reported by staff working on units in the active arm of the trial at 12 months, are interesting and raise the possibility that membership of peer-review networks could have costs as well as benefits for staff. Qualitative interviews conducted among staff on inpatient units that are part of peer-review networks study examining the process and outcomes of peer-review networks for inpatient mental health services reported that staff in the first year of membership felt anxious about their work being reviewed by their peers [38]. Unlike the inspections conducted by statutory authorities, the peer-review network’s aim is to promote a culture of openness and peer-support. However, it is possible that staff found it difficult to understand what and how networks operate in their first year of participation, which left them feeling under pressure to perform and under scrutiny by their peers [38].

In contrast to most of the previous literature on peer-networks, which has examined the impact of accreditation, teams that took part in this network were provided with feedback but were not required to demonstrate that they had acted on this in order to be ‘accredited’. It is possible that the additional requirement on services to demonstrate their performance against standards that is central to accreditation services provides a greater incentive for improvement than occurs in networks of the type we evaluated in this study.

Overall our study shows that experimental studies of peer-led quality improvement initiatives are feasible, but that longer follow-up periods may be needed to examine their impact. Randomised trials alone are not sufficient to explore the many factors that may affect quality of care and a mix-design study with quantitative and qualitative components may provide a better approach to understanding the complex processes and outcomes of peer-review networks.

## Conclusion

We conducted a cluster randomized controlled trial of a quality improvement peer-review network for low-secure mental health services to examine the impact of the network’s membership on the process and outcomes of care over a 12 month period relative to non-member services. The services that participated in the peer-review process showed a trend towards greater improvement in the quality of the physical environment relative to those that did not take part. However, this difference was not statistically significant. No differences were observed with regard to the patient-level outcomes at 12 months. The data collected form staff working in the service members of the network indicated that they felt safer on the ward compared to those working in the control services. However, they also experienced higher levels of burnout. A previous study indicated that during their first year of participation, staff in the service members often feel under pressure to perform and under scrutiny by their peers. We hypostasised that this may be associated to their higher levels of burnout observed in our study. Two main limitations of our study may have reduced our ability to demonstrate the impact of peer-networks on the outcome measures examined. Firstly, it was not possible to compare patient mental health and satisfaction and staff burnout at an individual level. Therefore, the results observed on staff and patient outcomes may reflect differences in the makeup of these groups between baseline and follow-up, rather than real changes associated to network’s membership. Secondly, we were only able to examine the impact of the first year of network membership which might be too short to observe evidence of service improvement. Future studies should examine longer term outcomes of participation in peer-review quality networks as well as in accreditation schemes which provide a greater incentive to improve quality relative to quality networks. In addition, using a mix methods design approach may provide a better understanding of the different factors that can affect quality improvement in health care and how quality networks operate.

## Abbreviations

CCQI: College Centre for Quality Improvement
COPD: Chronic Obstructive Pulmonary Disease
MBI: Maslach Burnout Inventory
MSU: Medium Secure Mental health services
PSQ: Patient Satisfaction Questionnaire
QELS: Quality of Environment in Low Secure Services checklist
SWEMWBS: Short Warwick-Edinburgh Mental Well-being Scale
